# Influence of Drugs and Toxins on Decomposition Dynamics: Forensic Implications

**DOI:** 10.3390/molecules29225221

**Published:** 2024-11-05

**Authors:** Shuyue Li, Zhonghao Hu, Yuming Shao, Guoan Zhang, Zheng Wang, Yadong Guo, Yu Wang, Wen Cui, Yequan Wang, Lipin Ren

**Affiliations:** 1 School of Forensic Medicine, Jining Medical University, Jining 272067, China; lsy9sunny@163.com (S.L.); shopsym@hotmail.com (Y.S.); zga2007@126.com (G.Z.); cuiwenmdd@163.com (W.C.); 2 Department of Forensic Medicine, School of Basic Medical Sciences, Xinjiang Medical University, Urumqi 830011, China; 3 Center of Forensic Science Research, Jining Medical University, Jining 272067, China; huzhonghao2020@163.com; 4 School of Electrical and Information Engineering, Hunan University, Changsha 410082, China; wangzheng@hnu.edu.cn; 5 Department of Forensic Science, School of Basic Medical Sciences, Central South University, Changsha 410013, China; gdy82@126.com; 6 Department of Forensic Medicine, Soochow University, Suzhou 215006, China; yuw@suda.edu.cn; 7 Precision Medicine Laboratory for Chronic Non-Communicable Diseases of Shandong Province, Jining 272067, China

**Keywords:** forensic toxicology, forensic entomotoxicology, metabolomics, microbiomics

## Abstract

Drug and toxin-related deaths are common worldwide, making it essential to detect the postmortem concentration of various toxic substances at different stages of decomposition in a corpse. Indeed, determining the postmortem interval (PMI) and cause of death in an advanced stage of decomposed corpses has been a significant challenge in forensic investigations. Notably, the presence of drugs or toxins can have a significant impact on the microbial profile, potentially altering the succession of microbial communities and subsequent production of volatile organic compounds (VOCs), which, in turn, affect insect colonization patterns. This review aims to highlight the importance of investigating the interactions between drugs or toxins, microbial succession, VOC profiles, and insect behavior, which can provide valuable insights into forensic investigations as well as the ecological consequences of toxins occurring in decomposition. Overall, the detection of drugs and other toxins at different stages of decomposition can yield more precise forensic evidence, thereby enhancing the accuracy of PMI estimation and determination of the cause of death in decomposed remains.

## 1. Introduction

According to the World Drug Report, approximately 450,000 people died from drug-related causes in 2015. Of these, 167,750 were directly attributed to drug use disorders, primarily due to overdoses, with opioids accounting for 76% of these cases. By 2022, nearly 292 million people, or 1 in 18, were globally reported to have used drugs. Cannabis remains the most widely used drug worldwide, with an estimated 228 million users, followed by opioids with 60 million. Amphetamine-type stimulants are used by 30 million people, while cocaine and “ecstasy” have 23 million and 20 million users, respectively [1]. The expanded range of drugs available to consumers has led to increasingly complex patterns, with polydrug use becoming a common feature across most drug markets. Additionally, the World Health Organization (WHO) recognizes suicide as a public health priority, with over 700,000 deaths attributed to suicide each year. Approximately 20% of global suicides are estimated to result from pesticide self-poisoning, particularly in the West Pacific region and developing countries in Asia and Africa [2,3]. It is well-known that the presence of drugs—whether prescribed or illicit—or other toxins in a deceased individual can affect the rate and pattern of decomposition, which can substantially impact forensic evidence interpretation [4]. Nonetheless, these effects remain relatively unexplored, potentially leading to inaccuracies and unquantifiable errors in the postmortem interval (PMI) estimation [5].

Generally, early postmortem changes, such as corpse temperature, ocular changes, the presence and distribution of muscular rigidity, the shape and extent of livor mortis, as well as the status of food in the digestive tract during autopsy, can be used to estimate the PMI [6]. Although these indicators offer valuable insights for estimating the PMI, the accuracy can vary significantly as decomposition is a complex biological and chemical process, along with individual differences among the deceased and environmental conditions [7,8]. Subsequently, with the rapid development of modern molecular biology, various biochemical changes, including shifts in electrolyte concentrations, enzyme activities, and the degradation of DNA, RNA (mRNAs, miRNAs, lncRNAs, circRNAs), and protein [9,10,11,12], have been used as potential biochemical markers for PMI estimation, making them valuable in criminal investigations [13]. Additionally, numerous studies have also focused on the biochemical changes in blood and other body fluids after death, which occur due to the absence of oxygen, alterations in enzymatic reactions, cellular autolysis, and the cessation of synthetic pathways [11,14]. These changes can be analyzed through metabolomics to investigate the metabolites that emerge after death, offering significant potential for identifying biomarkers in cases of poisoning [15]. For example, Donaldson and Lamont identified 26 metabolites, including 18 amino acids, glutathione (GSH), 4-Amino-n-butyric acid (GABA), glyoxylate, oxalate, hydroxyproline, creatinine, a-ketoglutarate and succinate, which showed increased concentrations post-mortem, and then proposed as potential markers for estimating the PMI [16]. Similarly, Dai et al. investigated the metabolic profiling of blood samples in rats poisoned with dichlorvos (DDVP) up to 72 h after death using GC/MS, indicating that a support vector regression (SVR) model constructed from 23 metabolites demonstrated significant potential in estimating the PMI in DDVP-poisoned rats [14].

As decomposition progresses, anaerobic bacteria, primarily in the gut, produce gas causing bloating, followed by the release of fluids and the subsequent dispersion of nutrients and microorganisms into the surrounding soil [17]. This microbial activity suggests that microbes can serve as valuable physical evidence in forensic investigations, as the succession of microbial communities occurs in a relatively predictable manner [18]. Indeed, previous studies have demonstrated repeatable shifts in the microbial community composition during the decomposition of terrestrial mammals, affecting cadaver skin, anus, mouth, nasal, eye, and ear cavity, internal organs, bone, and cadaver-related soils [17,19,20,21,22,23,24,25,26,27]. Moreover, age-related human microbiome dynamics undergo significant changes throughout life in response to various environmental and individual factors, including complex interactions between microbial functions, gastroenterological disorders, and therapeutic drugs [28,29]. As we all know, decomposition is a dynamic ecological process influenced by various factors, including environmental conditions, microbial activity, insect presence, vertebrate interactions, and the inherent characteristics of the individual [22]. When a corpse is recovered in an advanced stage of decomposition, particularly in cases involving skeletonized remains, it may be difficult to ascertain the cause of death, especially if the individual died from drug abuse or suicide in an isolated location [30]. In such cases, insects are of forensic importance as alternative toxicological samples. During the feeding process on cadaveric tissue, xenobiotics such as drugs and other toxic substances are transferred to the larvae and can also propagate through the food chain into other arthropods. Until now, the detection of drugs and toxins in insects has been documented [31]. Previous studies have also established a potential correlation between the concentration of drugs present in the substrate and developmental stages of insects [32,33,34]. Additionally, the developmental patterns of immature insects take place in a predictable manner under a controlled temperature, so the age determination of necrophagous flies is one of the key tasks in providing evidence for PMI estimation of decomposed corpses [35].

**Figure 1:**
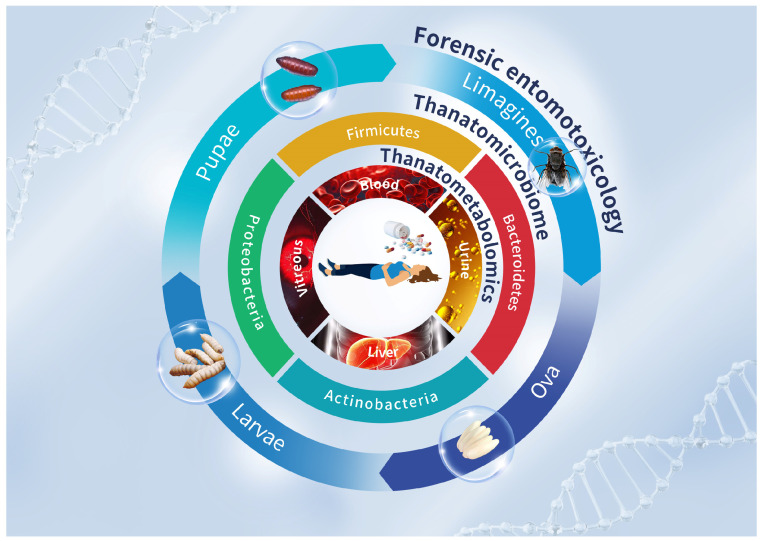
Overview of the detection of drugs and toxic substances in a deceased individual. Circular landscape from inner to outer: at the early PMI, thanatometabolomics allows for the detection of drugs and toxins in body fluids and tissues, such as blood, urine, vitreous humor and liver. As decomposition advances, the succession of microbial communities and necrophagous flies can be analyzed to provide insight into the presence of drugs or toxins.

In this review, we retrospectively analyzed the common types of drugs and toxic substances in poisoning cases, as well as their effects on cadaveric changes. Then, at the early PMI of a deceased individual, we can provide valuable clues for determining the time and cause of death through the combined observation of cadaveric phenomena and the detection of toxins, as well as their metabolites in body fluids and tissues. When a corpse is in an advanced stage of decomposition, the succession of microbial communities, volatile organic compounds (VOCs) production and necrophagous flies, along with the physiological and biochemical changes in the flies, can provide new insights into the detection of toxicants (Figure 1). Ultimately, the detection of drugs and other toxins at different stages of decomposition can yield more precise forensic evidence, thereby enhancing the accuracy of PMI estimation and cause of death determination in decomposed remains.

## 2. Materials and Methods

Articles were selected from the PubMed and Web of Science databases. The combined search terms: “forensic toxicology” + “metabolomics”, “forensic toxicology” + “PMI”, “forensic toxicology” + “microbial communities”, “microbial communities” + “PMI”, “microbial communities” + “VOCs”, “forensic entomotoxicology” + “PMI” were searched separately, yielding a total of 200 articles. An additional 165 relevant articles were identified through the references cited in these papers.

## 3. Advances in the Detection of Drugs and Toxic Substances in Corpses

The abuse of illicit drugs, such as cocaine and methamphetamine, presents significant socio-economic problems and is responsible for more than 200,000 deaths worldwide [1,2]. Meanwhile, sedatives have been implicated in numerous suicide cases globally due to overdosage [1]. In recent years, global antibiotic usage has surged by approximately 46% [36]. An upward trend in the use of antibiotics has led to overuse and subsequent resistance, highlighting the urgent need for heightened awareness and intervention regarding this issue. Moreover, environmental pollution, particularly the contamination of ecosystems with heavy metals, poses significant toxic risks to both flora and fauna, making it one of the most critical challenges facing the planet today [37]. Human samples, extensively examined for metabolic phenotyping, have emerged as a promising assay material with the potential to address critical questions, such as determining the cause of death by analyzing the concentrations of various biomarkers, which serve as practical and objective indicators. Furthermore, these samples offer valuable insights into the cellular changes that occur at the time of death, thereby assisting in the estimation of PMI [38]. For example, Pesko et al. [39] conducted a study comparing human and rat postmortem tissue, identifying threonine, tyrosine, and lysine as potential PMI markers. Similarly, Jawor et al. [40] identified six metabolites in plasma and urine that increased in levels with increasing PMI among a group of stillborn calves, further demonstrating the potential of these biomarkers. However, postmortem drug concentrations in a corpse can vary depending on the type of tissue and its location. These concentrations may also differ from those at the time of death due to postmortem redistribution, which can occur through the passive release of drugs from reservoirs in the gastrointestinal tract, lungs, and myocardium or from cell autolysis during advanced decomposition [41]. Therefore, it is crucial to detect the postmortem concentrations of various drugs and toxins at different stages of decomposition, as well as in diverse body fluids and tissues. Additionally, metabolomics presents an alternative strategy with the potential to detect biomarkers indicative of drug consumption [42].

At present, metabolomics analysis can be conducted using nuclear magnetic resonance (NMR) or mass spectrometry (MS) combined with separation techniques, such as liquid chromatography (LC), gas chromatography (GC), or capillary electrophoresis (CE) [15,43]. GC-MS is particularly effective for measuring volatile metabolites [44]. CE-MS is beneficial for detecting polar and ionogenic metabolites [45], while LC-MS can be utilized to analyze both polar and nonpolar metabolites [46]. Although NMR generally exhibits lower sensitivity compared to MS, it offers superior reproducibility and requires minimal sample preparation. NMR is inherently quantitative and considered a nondestructive technique [47]. Therefore, integrating NMR spectroscopy with ultra-performance liquid chromatography coupled with quadrupole time-of-flight mass spectrometry (UPLC-QTOF-MS) can significantly enhance analytical outcomes [48]. Additionally, the use of high-resolution mass analyzers, such as orbitrap or time-of-flight (TOF), MS is still essential for obtaining precise measurements in untargeted studies. Furthermore, fluorescent-based bioprobes have been developed to create highly sensitive nanobiosensors capable of detecting various biological and chemical agents [49]. These sensors offer several advantages over traditional analytical techniques, including GC, LC, and CE, due to their biodegradability, eco-friendliness, and greater operational and cost-effectiveness [50]. Previous studies have also demonstrated the effectiveness of the early detection of biomarkers associated with drug-induced organ damage [51]. In this review, we retrospectively analyzed the use of various analytical methods for detecting common toxins or drugs. Mainly focusing on substances such as sedatives, organophosphates and other pesticides, psychoactive drugs, heavy metals, ethanol, and common gas poisons, etc. (Table 1).

### 3.1. Detection of Pesticides and Their Metabolites in Corpses

Ingestion of pesticides is one of the most common methods of suicide worldwide, with organophosphates and carbamates being particularly significant causes of both accidental and intentional poisoning [1,77]. Recently, metabolomics has been applied to investigate the changes in metabolites following the administration of poisons, including DDVP [14,78], paraquat [79] and brodifacoum [80]. These studies suggest that toxicants can disrupt normal homeostasis, leading to alterations in antemortem metabolites, and may also influence postmortem metabolic shifts. For example, Gu et al. [81] explored whether acute DDVP poisoning could induce neurotoxic injury in the cerebrum and alter the expression of apoptosis-related genes in broilers, revealing that DDVP can cause the lysis of cerebrum nerve cell nuclei, complete destruction of the mitochondrial structures, changes in the expression of *ACC*, *LKB1*, *GPAT*, *HMGR*, *PPARα*, *CPT1* and *AMPKα1*, enhanced cell apoptosis, and significant neurogenic injury to the cerebrum [81]. Huang et al. [82] further explored the effects of acute DDVP poisoning on brain tissue in broilers, indicating that it could cause hyperglycemia, oxidative stress, brain edema, abnormal expression of glial fibrillary acidic protein (*GFAP*) and neuronal mitochondrial damage. Additionally, DDVP exposure significantly altered neurotransmitter secretion, energy metabolism, amino acid metabolism and nucleotide metabolism.

### 3.2. Detection of Common Gas Poisoning in Corpses

Carbon monoxide (CO) poisoning is a major cause of poisoning worldwide and represents a significant public health issue [83]. To assess the severity of CO poisoning, carboxyhemoglobin (COHb) concentrations in the blood are typically measured and correlated with autopsy findings [84]. However, recent studies have disputed the accuracy and specificity of COHb as a biomarker for antemortem CO exposure and proposed total blood CO (TBCO) as a novel and potentially more reliable biomarker [73,85]. TBCO has shown promising results in improving the accuracy of CO detection in blood and has demonstrated greater stability under various storage conditions [73]. Recently, Oliverio et al. [86] measured postmortem TBCO concentrations from different autopsies blood samples, including cardiac, peripheral, spleen and cranial blood. The study established reference values for TBCO that could help differentiate antemortem CO exposure, further suggesting that TBCO may be a more effective biomarker for CO poisoning compared to COHb.

In addition, Hydrogen sulfide (H_2_S) is one of the most serious toxic gases and a common cause of fatal workplace accidents [87]. However, direct H_2_S detection rarely allows us to determine whether the cause of death resulted from antemortem exposure to the gas [88]. Previous studies have suggested that thiosulfate, a stable and reliable biomarker, can be detected in postmortem blood and urine to distinguish between H_2_S poisoning and putrefactive phenomena, and which can also provide insights into the chronological order of deaths in cases involving multiple fatalities [89]. For example, Ventura et al. [74] reported a severe case of accidental H_2_S poisoning involving six sailors, in which thiosulfate levels were crucial in distinguishing whether the deaths were due to H_2_S inhalation or decomposition processes. The individuals who survived the longest had the highest concentration of thiosulfate, while those who died sooner had lower concentrations. This study highlights the utility of thiosulfate as a forensic marker in evaluating H_2_S poisoning, offering insights into survival time and event dynamics [74]. Besides, liquefied petroleum gas (LPG) mixtures are commonly involved in suicides, with ethanethiol being a frequent component. Aquila et al. [90] presented a case of LPG poisoning and suggested that in a similar case of acute LPG inhalation, ethanethiol may accelerate the process of decomposition, potentially leading to inaccurate PMI estimation. Therefore, the detection of common volatile gases in corpses is crucial in forensic toxicology. It requires precise and sensitive analytical methods to identify and quantify these compounds, which are essential for determining the cause of death. Understanding the specific properties and risks associated with each substance is vital for accurate diagnosis and legal proceedings.

### 3.3. Detection of Alcohol and Its Metabolism in Corpses

A new report from the WHO highlights that 2.6 million people die per year from alcohol consumption, accounting for 4.7% of all deaths. An estimated 400 million people suffer from alcohol use disorders worldwide, with alcohol being a leading cause of death associated with traffic accidents, violent crime, and overdose [1]. So ethanol is the most commonly used psychoactive substance, but it can also be produced postmortem through putrefactive processes due to microbial activity [91]. It is crucial to determine whether the ethanol present in samples originated from antemortem consumption or was synthesized after death within the corpse [92]. Consequently, interpreting alcohol concentration at the time of death and linking it to the cause of death remains a well-known challenge in forensic toxicology. Blood, urine, and vitreous humor are commonly used for postmortem ethanol testing [93,94].

Previous studies have shown that even in cases of postmortem ethanol synthesis, the detection of Ethyl glucuronide (EtG) and ethyl sulfate (EtS) in cadaveric biological fluids indicates antemortem alcohol consumption [95], which is stable across various storage temperatures [96], making them reliable biomarkers for monitoring chronic alcohol abuse [97]. EtG and EtS have distinct toxicokinetic profiles, characterized by a significantly slower degradation rate [98], which may also hold great potential in estimating the PMI in cases of alcohol-related deaths. Forensic toxicologists have developed ethanol biomarker-based approaches to aid in such assessments [94]. For example, Al-Asmari et al. [71] investigated postmortem ethanol biomarkers, EtG and EtS, under various conditions, including diabetes mellitus, drug abuse, and advanced decomposition, and found a significant positive correlation between EtG/EtS levels and known alcoholic individuals, enabling accurate identification of ethanol sources. Additionally, the PMI and ambient temperature significantly impact postmortem ethanol production [71]. However, most current studies have focused on body fluids such as vitreous humor. In cases where these fluids are unavailable, solid tissues may be preferred for detecting ethanol biomarkers, especially in advanced decomposition.

### 3.4. Detection of Other Uncommon Toxins and Their Effect on PMI Estimation

Cantharidin poisoning is rare but potentially fatal in clinical practice, particularly in patients who have used it irrationally as an alleged aphrodisiac [99] or in cases of accidental poisoning due to the ingestion of blister beetles [100]. Many patients exhibit no obvious symptoms in the early stage of cantharidin poisoning, but most of them have poor prognoses once diagnosed with acute circulatory failure. Although clinically uncommon, cantharidin poisoning can be fatal, particularly when used improperly [101]. As a result, homicidal cases involving cantharidin poisoning can be difficult to detect, posing significant challenges to forensic investigations. The detection of postmortem cantharidin concentration is crucial in cases of suspected poisoning. Recently, Zhang et al. [102] developed a rapid and sensitive LC-MS/MS method for quantifying cantharidin in the liver and kidney of rats and found that the highest concentration of cantharidin occurred 72 h after death, which may be related to postmortem metabolism and decomposition processes. This study also suggests that the determination of cantharidin in the liver of rats has great potential for PMI estimation [102]. However, the mechanisms behind postmortem changes in cantharidin concentration remain unclear. Furthermore, myocardial injury induced by cantharidin is one of the most severe organ damage, depending on the dose and duration of exposure [103]. Subsequently, Zhang et al. [104] further demonstrated that plasma levels of troponin T (TN-T) and the HIF-1α/TN-T ratio are highly accurate cardiac biomarkers for postmortem diagnosis of cantharidin-induced myocardial injury, and VEGF/HIF-1α ratio is proposed as a potential biomarker for PMI estimation.

## 4. Effects of Drugs and Toxins on Development of Necrophagous Flies

Indeed, determining the PMI and cause of death in decomposed corpses has been a significant challenge in forensic investigations. As a corpse decays, it undergoes a wide range of biological and chemical changes that can obscure crucial shreds of evidence, making it difficult to ascertain. Putrefied tissues, influenced by various biological and environmental factors, are generally not suitable for standard chemical analysis [105,106]. This is particularly acute in cases where corpses are found in advanced stages of decomposition, such as those involving drug abuse in isolated areas [31]. In such scenarios, forensic entomotoxicology is valuable for conducting drug analyses when samples of tissues, blood, or urine cannot be extracted from decomposed corpses [107], which has been extensively explored as an alternative method of detecting drugs and toxins (Table 2). It is widely known that toxic substances accumulated in fly larvae can positively or negatively influence the length and weight fluctuations as well as the typical duration of their life cycles [108], so these studies contribute to standardizing methodologies and data for PMI estimation, ensuring that they meet the Daubert criteria for the admissibility of scientific evidence in court.

Since then, a wide range of drugs and toxic substances have been detected in the larvae of different species, mainly including Amitriptyline, Propoxyphene, Acetaminophen [124], Steroids [125], Trazodone, Trimipramine and Temazepam [126], Barbiturate and Meprobamate [127], Methylphenidate [128], Heroin [129], Cocaine [130], Benzodiazepine [131], Codeine [132], Alcohol [133], as well as Aluminium phosphide and Diazinon [33,134,135], Terbufos [136], Cypermethrin [137], etc. Furthermore, when larvae actively feed on poisoned corpses, xenobiotics, such as drugs and other toxins present in the tissues, are transferred into their metabolic systems. These substances can accumulate inside the larvae’s cuticle during growth and may be preserved within the sclerotized puparium during pupariation. Therefore, puparia serve as a crucial resource for toxicological samples when corpses are recovered in a skeletonized stage. Besides, various terrestrial arthropods are also attracted by the odors emitted from decomposed corpses. It is also important to note that toxicants can significantly prolong the pre-appearance interval (PAI) of insects and delay oviposition [5]. Investigating the impact of PAI and oviposition on decomposition rates, microbial succession, and VOC production can shed light on the role of toxicants as drivers of cadaveric changes. By incorporating these factors, such as toxic substances and environmental factors, into the core, we can achieve a more comprehensive understanding of the complex ecological processes that occur during decomposition.

## 5. Advances in Microbial Communities to Estimate PMI in Poisoned Corpses

The human microbiome plays a crucial role in the decomposition process and significantly influences the interactions between insects and their surrounding environment. This makes it a potential tool for estimating the PMI [138,139]. Necrobiome is generally explored for microorganisms colonizing on the surface and orifice [140], body fluids [141], and internal organs of corpses [142]. Currently, universal genetic markers such as 16S rRNA, 18S rRNA, and ITS gene regions are used to capture a snapshot of the microbial taxa present in a sample, providing a rich dataset for taxonomic analysis. During decomposition, the dominant bacterial phyla in the human gut are mainly represented by *Firmicutes*, *Bacteroidetes*, *Actinobacteria*, *Proteobacteria*, and *Verrucomicrobia* [24,143]. Notably, the relative abundance of *Proteobacteria* increased significantly, while *Firmicutes* and *Bacteroidetes* gradually decreased over time of death, which are crucial for estimating PMI [144,145]. Meanwhile, Adserias-Garriga et al. also depicted the thanatomicrobiome successional changes in the oral cavity of three human cadavers [146], indicating that *Firmicutes* and *Actinobacteria* were the predominant phyla in the fresh stage. Subsequently, during the bloat stage, the dominant families included *Peptostreptococcaceae* and *Bacteroidaceae*, along with a relatively high abundance of *Ignatzschineria*, which is associated with parasitism of larvae in the Sarcophagidae family. These findings are almost consistent with previous studies indicating that *Bacteroidales* (*Bacteroides*, *Parabacteroides*) significantly declined in the human gut bacterial communities [21,147], while *Clostridiales* (*Clostridium*, *Anaerosphaera*) and the fly-associated Gamma-*proteobacteria* (*Ignatzschineria* and *Wohlfahrtiimonas*) increased [21]. During the advanced decay stage, the predominant bacterial families included Gamma-*proteobacteria*, *Pseudomonadaceae*, *Alcaligenaceae*, and *Planococcaceae* [23,146]. Notably, Gamma-*proteobacteria* and *Proteus* within the oral bacterial community showed a significantly positive correlation with the PMI during the later stage of decomposition in mice. Furthermore, dry remains were primarily characterized by the presence of *Bacilli* and *Clostridia* [146].

Moreover, the microbiota of living hosts is highly influenced by various factors, including the environment, the presence or absence of disease, and the use of illicit or prescribed chemical substances [148]. For example, Jackson et al. conducted an analysis of gut microbiota associations with 38 common diseases and 51 medications, revealing that both paracetamol and opioids are linked to a higher abundance of *Streptococcaceae* [149]. This demonstrates that the presence of drugs or toxins can significantly alter the composition of microbial communities. Additionally, environmental factors are crucial when estimating PMI, as the microbiome is affected by temperature, humidity, and other environmental conditions [18]. Consequently, microbial succession during decomposition should be evaluated across various seasons, geographic regions, and locations (e.g., indoors, outdoors, underwater, buried or burned remains). Similarly, given the significant interindividual differences in the human microbiome, which can be influenced by factors such as diet and the antemortem environment, it remains necessary to investigate whether these variations impact PMI estimation [150,151]. Fortunately, machine learning methods have been developed to create regression models for estimating PMI [152,153]. These microbiome-based models have provided accurate PMI estimation within certain ranges, accompanied by quantifiable error rates.

## 6. Interdependence of Postmortem Microbes and VOCs

The attraction and colonization of vertebrate remains due to carrion-related arthropods, which are primarily governed by olfaction. As a corpse decomposes, microbial activity leads to the release of VOCs that serve as chemical signals to attract insects [154]. The composition and concentration of VOCs are influenced by the presence and abundance of specific microbial species and arthropods [155]. The core VOCs in decomposition odor have been shown to change predictably over time, and this temporal variation can be developed into biochemical markers to estimate the PMI [3,156]. Additionally, VOCs can provide insights into the prior location of decomposed remains [157,158,159], highlighting their great potential in forensic investigations. While elucidating the temporal trends of individual VOCs can enhance our knowledge of overall changes in the composition of VOCs during decomposition. Combining bacterial species monitoring with VOC production could significantly strengthen our understanding of these mechanisms. To date, primary studies have seldom focused on the specific relationship between postmortem microbes and their role in the emission of volatiles. Fortunately, ongoing research has improved our understanding of VOCs released from individual microbes or microbial communities [160]. For instance, VOCs can indicate the presence of specific bacterial species within a microbial community [161] and serve as indicators of bacterial behavior and life cycles, including how these are influenced by interactions with other species [162,163]. Therefore, an increasing number of foundational studies linking postmortem bacteria and VOCs could greatly enhance our ability to explore VOCs as forensic traces, providing potential tools for PMI estimation.

It is well established that microorganisms play a dominant role in the production of VOCs through their interactions with cadaveric nutrients and the release of volatile by-products from their own metabolism [160,164]. At present, although these multidisciplinary studies involving fieldwork trials have been limited, the data available on the relationship between bacteria and VOCs offer promising insights into linking the microbiology and chemistry of human remains. For example, Pascual et al. [164] showed that individual VOCs were correlated with specific taxa during the early stages of decomposition, which are primarily dominated by bacteria. Through best-fitting multiple linear regression models, the synthesis of acetic acid, indole and phenol could be linked to the activity of *Enterobacteriaceae*, *Tissierellaceae* and *Xanthomonadaceae*, respectively [164]. Cernosek et al. [165] established the VOC profile released by three postmortem bacterial isolates (*Bacillus subtilis*, *Ignatzschineria indica*, *I. ureiclastica*), indicating that each bacterial species exhibited a distinct VOC profile. Notably, *I. indica* produced significant amounts of 3- methyl-2-pentanone and phenol in its temporal profile [165]. Subsequently, Furuta et al. [163] further characterized the VOCs released by five postmortem bacterial species (*B. subtilis*, *I. indica*, *I. ureiclastica*, *Curtobacterium luteum*, and *Vagococcus lutrae*), identifying five VOCs that were consistently found across these different bacterial species, including acetone, dimethyl disulfide, dimethyl trisulfide, 2-pentanone, and 1-butanol. Interestingly, dimethyl disulfide was observed at relatively consistent levels across different bacterial species and is one of the most consistently identified compounds in decomposition odors [163].

Therefore, the abundance of several postmortem VOCs is not random; it is significantly correlated with bacterial taxa. If the functional activity of microorganisms responsible for releasing VOCs can be identified, it will contribute to clarifying the source of chemical components in decomposed odors. A recent study linked bacteria families to VOCs detected in pig carcasses [162], showing relatively consistent levels of dimethyl disulfide across 0–4 days, which also coincided with the presence of *Enterococcaceae* and *Bacillaceae*. Meanwhile, Furuta et al. also found that *V. lutrae* and *B. subtilis*, belonging to these same families, were aligned with this timing and VOC trends [163]. Previous studies have provided foundational data linking decomposition odor to specific postmortem microbes and explaining VOC trends through the establishment of different bacterial species at various stages of decomposition. Specifically, further studies should aim to pinpoint individual VOC trends to groups of bacterial species, ultimately enhancing our understanding of the underlying mechanisms responsible for the decomposition of VOC production. This could significantly improve the accuracy and reliability of VOCs as potential biomarkers in forensic investigations.

## 7. Implications and Conclusions

In cases where poisoning is suspected as the cause of death, various body fluids and tissues can be directly extracted and chemically analyzed during the early PMI. At the same time, metabolomics plays a crucial role in identifying biomarkers indicative of drug or toxin exposure and can be further enhanced by highly sensitive, targeted analyses such as nano-biosensors for rapid drug and toxin detection. As decomposition progresses, drugs and toxins can substantially impact microbial profiles, altering microbial succession and influencing the production of VOCs, which in turn affect insect colonization patterns. Additionally, advances in artificial intelligence, particularly deep learning, offer significant potential to integrate and analyze multi-omics data, enhancing the detection of toxic substances. Therefore, this review investigates the interactions between drugs or toxins, microbial succession, VOC profiles, and insect behavior, providing valuable insights into forensic investigations and the ecological impact of toxins during decomposition.

## Figures and Tables

**Table 1:** Overview of metabolites as the potential biomarkers of drug/poison in testing samples.

Classification	Drugs/Toxins	Analytical Methods	Samples	Sample Preparation	Metabolite/Biomarkers	References
Psychoactive substance	Methamphetamine (METH)	LC-MS/MS	blood	300 μL acetonitrile with 0.1% formic acid and 100 μL IS solution were added into the plasma sample.	proline, serine, alanine, biotin, and nicotinamide	[52]
		GC-MS	serum and urine of mice	methanol solution with internal standard compound was added to the samples to precipitate protein. Methoxyamine and a trimethylsilanization reagent was added. The external standard methyl myristate in heptane was added.	palmitic acid, 5-hydroxytryptamine, monopalmitin, and phenylalanine	[53]
		HPLC-MS/MS	hair	air strands from the root were finely cut. Hair segments were incubated in methanol. The extract was evaporated to dryness and the residue was reconstituted in methanol.	glycosphingolipids, sphingolipids, glycerophospholipids, glycine, serine and threonine, cysteine and methionine	[54]
	Benzofuran	LC-HR-MS	rat urine	Samples were extracted after conjugate cleavage with a mixture of glucuronidase and arylsulfatase by solid-phase extraction. Afterwards, the residue was reconstituted in methanol.	3-carboxymethyl-4-hydroxy amphetamine	[55]
	Morphine	LC-MS	male mice	Samples were reconstituted in solvent containing methanol. A 100 µL sample was added to extraction solvent and reconstituted in reconstitution solvent.	riboflavin and flavonoids	[56]
	Heroin/Morphine	LC-MS/MS	vitreous humor	Sample was diluted with 150 µL equine plasma, and then spiked with 50 µL of Md3 and M3Gd3 (IS). After vortexing, 1 mL of 0.5 M Ammonium Carbonate solution was added.	6-monoacetylmorphine (6-MAM)	[57]
	Heroin	LC-MS/MS	rabbit blood	Samples were homogenised with two parts of methanol and four parts of hexane. The methanol/hexane homogenates were then centrifuged, and the top hexane layer discarded.	morphine and morphine-3-glucuronide	[58]
	Tramadol	GC/MS	bone marrow and fly larvae	A deproteinization mixture of acetone and benzyl-alcohol was added in samples, and then samples were extracted with diethyl ether.	caffeine and N-desmethyltramadol	[59]
	Barbiturates (arbital, phenobarbital, and pentobarbital)	FM-LPME/LC-MS	human blood, urine, and liver	The blood sample was diluted using 10 mM HCl solution. The urine sample was diluted containing the three barbiturates at the desired concentration and IS. m liver tissue was weighed and homogenized after overnight lyophilization.	/	[60]
	amphetamines, ketamine	LC-MS	hair	hair was ground with 2 mL of saturated sodium carbonate solution using a high-efficiency hair grinder. After centrifuging, 1.5 mL of the supernatant was transferred and treated with solid-phase microextraction (SPME) by direct immersion (DI-SPME).	/	[61]
	Methadone, Buprenorphine, Oxycodone, Fentanyl	UHPLC–MS-MS	Pericardial fluid (PF), muscle and vitreous humor (VH)	PF and VH were sampled in evacuated tubes, with 20 mg of sodium fluoride and heparin. Muscle samples were homogenized in water.	/	[62]
	dextro- (DXM) and levo-methorphan (LVM)	LC–MS/MS	human blood	10 μL internal standard mixture and 2 mL chloroform mixture were added to whole blood. Samples were reconstituted in 100 μL methanol.	dextrorphan and levorphanol	[63]
	Methoxetamine (MXE)	LC-MS	human blood and urine	100 µL of the biological specimen sample and 600 µL water were added to acetonitrile, and then vortexed and centrifuged.	O-desmethyl MXE, O-desmethyl-hydroxy MXE, N-desethyl-O-desmethyl MXE sulfate, O-desmethyl MXE glucuronides and sulfates	[64]
	Cannabis	LC-MS/MS	urine, bile, and gastric contents	200 µL of sodium hydroxide was added to the samples, and which further kept for 20 min in water bath before proceeding with the hydrolysis	11-nor-Δ^9^-tetrahydrocannabinol-9-carboxy (THC-COOH)	[65]
	Etizolam	LC–MS-MS	human blood	Samples were precipitated with acetonitrile. The supernatant was decanted into removal tubes. Extracts were reconstituted in mobile phase.	/	[66]
	54 benzodiazepines	UHPLC-QqQ-MS/MS	blood, urine, vitreous humor and bile	A 100 μL volume of biological fluid was extracted, 10 μL of methanolic internal standards mixture solution and 100 μL of 0.5 M ammonium carbonate buffer were added. Liquid-liquid extraction with 2 mL of ethyl acetate was carried out.	/	[67]
	79 new psychoactive substances (NPS)	UHPLC-MS/MS	blood and urine	500 µL of biological fluid and 20 µL of the IS mix were added. After adding water and CAN, 500 mg of a mixture of anhydrous MgSO_4_/NaOAc was added, and then reconstituted with mobile phase B.	/	[68]
	nine NPS	LC/ESI−MS/MS	dried blood spot	The samples were placed in microtubes, to which methanol-acetonitrile containing the IS was added. After evaporation, the resulting residue was redissolved in acetonitrile.	/	[69]
	Olanzapine	UHPLC-MS/MS	hair	Extraction medium, consisting of methanol, acetonitrile and ammonium formate were added with samples. Internal standard solution was added and the hair was pulverized. The extracts were diluted with water.	/	[70]
Pesticide	Dimethyl dichloroviny phosphate (DDVP)	GC-MS	blood of rat	Blood sample was added with cold acetonitrile. The resultant derivatives were rapidly added with N, O-bis-(trimethylsilyl)-trifluoroacetamide with trimethylchlorosilane.	valine, isoleucine and pyruvate	[14]
Alcohol	Ethanol	LC-MS/MS	human blood, urine, vitreous humor	Samples were centrifuged, supernatant was placed into a test tube containing internal standard and mixed with ice-cold methanol.	ethyl glucuronide (EtG), ethyl sulfate (EtS)	[71]
	Methanol	GC-FID	cartilage tissue	formic acid was determined in the form of a volatile methyl formate ester. Using the FID detector ensured the sensitivity of 0.01 mg/mL and reduced the impact of the biological background.	formic acid	[72]
Gas	Carbon monoxide (CO)	GC-MS	Blood	Blank bovine blood was added with the respective preservative immediately after collection. To ensure homogenization, the blood-containing bottles containers were agitated for 20 min.	carboxyhemoglobin (COHb) and total blood CO (TBCO)	[73]
	Hydrogen sulphide (H_2_S)	GC-MS	blood and urine	Blood and urine aliquots were added to a mixture of 0.5 mL of 20 mM pentafluorobenzyl bromide PFB-Br solution in toluene, 2.0 mL of internal standard solution and 0.8 mL of 5 mM TDMBA solution.	thiosulfate	[74]
Heavy metal	Lead (Pb), cadmium (Cd), and mercury (Hg)	UPLC-MS/MS	blood and brain of rats	Samples were prepared by treating serum with precooled methanol and acetonitrile. The metabolites were resuspended in 150 μL of 50% methanol and centrifuged for 30 min at 4000 rpm.	arachidonic acid, adrenic acid, kynurenic acid and quinolinic acid	[75]
Gabapentinoids	Pregabalin and gabapentin	LC-TOF-MS	blood and urine	The diluted samples were spiked with reference standardat concentrations. Protein precipitation was carried out by vortex mixing with trichloroaceticacid. The aqueous layer was transferred into anautosampler vial and diluted.	/	[76]

**Table 2:** Effects of drugs and toxins on the development of necrophagous flies.

Classification	Drugs/Toxins	Species	Baits	Developmental Rate	References
Pesticides	Aluminum phosphide (AlP)	*Chrysomya albiceps* (Wiedemann, 1819)	Rabbit	The developmental cycle increased significantly. Larval body was significantly smaller.	[109]
		*Chrysomya megacephala* (Fabricius, 1794) *Chrysomya rufifacies* (Macquart, 1843)	Rabbit	AlP accelerated development until pupation, and had a positive effect on the development at higher concentrations.	[33]
	Roundup Full^®^ II (herbicide)	*Lucilia sericata* (Meigen, 1826)	Pig	The duration of the developmental stages remained unchanged, but all size parameters of the puparium were reduced.	[110]
	Terbufos (Organophosphate)	*Lucilia eximia* (Wiedemann, 1819) *Peckia chrysostoma* (Wiedemann, 1830)	Rat	Larvae of *L. eximia* were more active, with greater frequency of body movements and lateral contractions. Immatures of *P. chrysostoma* were less active, with fewer body and lateral contractions.	[111]
	Malathion	*Megaselia scalaris* (Loew, 1866)	/	It reduced the larval growth rate and increased the duration of the larval stage.	[112]
Psychoactive drugs	Cocaine and heroin	*Calliphora vomitoria* (Linnaeus, 1758)	Pork	Cocaine shortened pupation and accelerated eclosion, as well as developed less in length and weight. Heroin led to lengthier pupation, showed a being smaller and lighter.	[34]
	Methamphetamine	*Calliphora stygia* (Fabricius, 1781)	Sheep’s liver	Larval growth significantly accelerated and increased the size of all life stages. The pupal stage was prolonged 78 h.	[113]
		*Sarcophaga ruficornis* (Fabricius, 1794)	Rabbit	The time required for pupariation was significantly longer.	[114]
		*Aldrichina grahami* (Aldrich, 1930)	Rabbit	The developmental time to reach the pupal stage was slower. the mean length of larvae was longer.	[115]
Sedative drugs	Zolpidem tartrate	*Chrysomya megacephala* (Fabricius, 1794) *Chrysomya saffranea* (Bigot, 1877)	Buffalo liver	The weight, length, and width decreased as the concentration increased. The duration of both developmental stages increased as the concentration increased.	[116]
	Lorazepam	*Chrysomya rufifacies* (Macquart, 1843)	Beef liver	Length, weight, and width of larvae decreased with increased concentration of lorazepam.	[117]
Antibiotics	Ciprofloxacin	*Sarcophaga peregrina* (Robineau-Desvoidy, 1830)	Pig lung	The length of larvae increased with higher drug concentrations, while the weight of both the pupa and adult decreased significantly.	[118]
	Ceftriaxone and levofloxacin	*Lucilia sericata* (Meigen, 1826)	Pork	The time to pupation was significantly extended, and the mortality rate increased.	[119]
		*Calliphora vomitoria* (Linnaeus, 1758)	Pork	The maggot growth was delayed by levofloxacin, but not with ceftriaxone. Pupation was delayed in both antibiotics, and mortality was reduced.	[120]
		*Protophormia terraenovae* (Robineau-Desvoidy, 1830)	Pork	The maggot development time was significantly decreased. The time to start pupation was significantly increased. The survivability of the maggots was improved.	[121]
Heavy metals	Cadmium Chloride	*Chrysomya megacephala* (Fabricius, 1794)	Rats	Development time was prolonged at higher concentrations, and larval mortality increased with the concentration.	[122]
	Cadmium, Zinc, Copper	*Lucilia sericata* (Meigen, 1826)	Chicken liver	Larval and pupal survival decreased as heavy metal concentrations increased. Pupal weight and larval length were significantly different among heavy metals and concentrations.	[123]

## Data Availability

No new data were created or analyzed in this study.
